# Association between smoking and height loss in Japanese workers: A retrospective study

**DOI:** 10.1371/journal.pone.0298121

**Published:** 2024-02-15

**Authors:** Yuji Shimizu, Nagisa Sasaki, Hidenobu Hayakawa, Eiko Honda, Midori Takada, Takeo Okada, Tetsuya Ohira

**Affiliations:** 1 Epidemiology Section, Division of Public Health, Osaka Institute of Public Health, Osaka, Japan; 2 Department of Cardiovascular Disease Prevention, Osaka Center for Cancer and Cardiovascular Disease Prevention, Osaka, Japan; 3 Department of Epidemiology, Fukushima Medical University School of Medicine, Fukushima, Japan; Hamasaki Clinic, JAPAN

## Abstract

Height loss is reported to be an independent risk factor for all-cause and cardiovascular mortality. Smoking, which is responsible for a considerable proportion of deaths due to any cause, is also associated with lumbar disc degeneration, a major risk factor for height loss. Therefore, smoking could be an independent risk factor for height loss. To clarify the association between smoking status and height loss, a retrospective study with 8,984 (5,518 men and 3,466 women) Japanese workers was conducted. The present study population comprised 9,681 workers aged 40–74 years who participated in annual medical examinations between 2011 and 2017 (baseline). Subjects without a height measurement during 2012–2018 (endpoint) were excluded from the analysis (n = 697). Height loss was defined as being in the highest quartile of annul height decrease (1.48 mm/year for men and 1.79 mm/year for women). Independent of known cardiovascular risk factors, smoking was positively associated with height loss among men but not among women. With never smokers as the referent group, the adjusted odds ratio (95% confidence interval) was 1.15 (0.98, 1.35) for former smokers and 1.24 (1.05, 1.46) for current smokers among men, respectively. Among women, the corresponding values were 0.98 (0.79, 1.21) and 0.90 (0.71, 1.16), respectively. Since height loss and smoking are independent risk factors for all-cause and cardiovascular mortality, these results help clarify the mechanisms underlying the association between height loss and mortality risk.

## Introduction

Height loss starting in middle age is reported to be a risk factor for all-cause and cardiovascular mortality in later life [1]. Even a small height loss is associated with a higher risk of all-cause mortality [2]. Furthermore, independent associations between the degree of height loss and incidence of cardiovascular disease have also been reported [3].

Smoking is also responsible for a considerable proportion of deaths due to any cause [4]. Smoking is positively associated with lumbar disc degeneration [5] and osteoporosis [6]. Since lumbar disc generation and vertebral fracture, which is strongly related to osteoporosis [7], are known major risk factors for height loss among adults, smoking could affect height loss in the general population.

However, smoking increases hemoglobin levels [5]. Our previous study revealed an inverse association between hemoglobin levels and height loss among male Japanese workers but not among female Japanese workers [8].

These studies indicated a contradiction in that smoking, which increases mortality [4], also increases hemoglobin levels [5] whereas height loss, which is associated with high mortality [1, 2], is inversely associated with hemoglobin levels [8].

Therefore, clarifying the association between smoking status and height loss could be help clarify the mechanisms underlying the association between mortality risk and height loss. Furthermore, clarifying the effect of hemoglobin on those mechanisms also might help with estimating the risk for height loss.

## Materials and methods

A retrospective study of 8,984 Japanese workers who participated in annual health check-ups at least twice between 2011 and 2018 was conducted to clarify the association between smoking status and height loss. The material and methods used in the present risk survey for height loss have been described elsewhere [8–10].

### Study population

The Ministry of Health, Labour and Welfare of Japan started specific medical examinations for cardiovascular disease prevention in 2008. In addition to physical examinations and general laboratory tests of blood and urine samples, the medical examination contained a questionnaire about lifestyle and medical history. In Japan, companies have an obligation to make their employees undergo specific medical examinations for cardiovascular disease prevention each year. In this study, a template of the questionnaire form recommended by the Japanese government was used.

The present study population comprised 9,681 workers aged 40–74 years who participated in these specific medical examinations between 2011 and 2017 (baseline) at the Osaka Center for Cancer and Cardiovascular Diseases Prevention. Since the participants of this study were workers who had the capacity to work, they might have been relatively healthier than the general population. Furthermore, compared to the general population, the proportion of men might be higher because men are more likely to work than women in Japanese society. This study was approved by the ethics committee of the Osaka Center for Cancer and Cardiovascular Diseases Prevention (project registration code: R4-Rinri-4). All procedures involving human participants were performed in accordance with the ethical standards of the ethics committee of the Osaka Center for Cancer and Cardiovascular Diseases Prevention and the 1964 Helsinki Declaration along with its amendments. Consent for this study was obtained using the opt-out method with descriptions of the study presented on posters and the institution’s website (www.osaka-ganjun.jp/effort/cvd/r-and-d/) accessed on 20 July 2023.

Since the present study used data on height decrease per year, at least two height measurements (at baseline and endpoint) during the observation period were necessary. Subjects without a height measurement during 2012–2018 (endpoint) were excluded from the analysis (n = 697). The remaining 8,984 subjects with a mean age of 50.6 years (standard deviation (SD), 8.3 years; range, 40–74 years) were included in the study. The mean follow-up period was 3.5 years (SD, 2.0 years; interquartile range, 1.9–5.7 years).

### Baseline data

The baseline period of the present study was 2011–2017. Trained interviewers acquired data on medication history and habits. Briefly, height in feet while wearing stockings and weight while wearing light clothing were measured. Body mass index (BMI) was calculated as weight divided by height squared (kg/m^2^). Resting blood pressure was measured twice and the average was used in the analysis. Before the second blood pressure measurement, participants were asked to take a deep breath.

A fasting blood sample was collected. Hemoglobin, total cholesterol (TC), triglycerides (TG), high-density lipoprotein cholesterol (HDLc), Hemoglobin A1c (HbA1c), and serum creatinine were measured using standard procedures at the Osaka Center for Cancer and Cardiovascular Diseases Prevention. Low-density lipoprotein cholesterol (LDLc) was calculated using the Friedewald formula: LDLc (mg/dL) = TC–(HDLc/5).

Between 2011 and 2012, HbA1c values were measured using the Japanese Diabetes Society (JDS) definition. Starting in 2013, HbA1c values were measured using the National Glycohemoglobin Standardization Program (NGSP) definition. The following equation, which was recently proposed by a JDS working group, was used to convert values: HbA1c (NGSP) = HbA1c (JDS) + 0.4% [11].

The present study attempted to evaluate the risk of height loss associated with smoking and clarify the potential mechanism underlying the associations among smoking, hemoglobin, and height loss. Therefore, multi-faceted analysis that evaluates the risk of height loss by hemoglobin level and smoking status were performed. In addition, hemoglobin levels could be influenced by smoking status [5] and age [12]. Correlations between hemoglobin and smoking status and between hemoglobin and age were also evaluated.

Overweight (BMI ≥ 25 kg/m^2^) is an independent risk factor for height loss [10]. Endothelial repair activity, as evaluated by circulating CD34-positive cell count [13], has been reported to be inversely associated with height loss [14]. Hypertension, which is known to be associated with endothelial dysfunction [15], is also positively associated with height loss [16]. Therefore, endothelial status, which is a cardiovascular risk factor, might play an important role in height loss. TG and HDLc are also associated with hypertension and endothelial repair activity [17, 18]. In addition, HbA1c, which is known as a marker of diabetes, is positively associated with height loss [9]. Therefore, BMI, hypertension, dyslipidemia, and diabetes, which are known cardiovascular risk factors, could act as confounders in an analysis evaluating the risk of height loss.

We defined high BMI as BMI ≥ 25 kg/m^2^ and low BMI as BMI <18 kg/m^2^. Hypertension was defined as systolic blood pressure ≥ 140 mmHg, diastolic blood pressure ≥ 90 mmHg, or use of anti-hypertensive medication. Dyslipidemia was defined as TG ≥ 150 mg/dL, LDLc ≥ 140 mg/dL, HDLc < 40 mg/dL, or use of lipid-lowering medication. Diabetes was defined as HbA1c (NGSP) ≥ 6.5% or use of glucose-lowering medication.

### Statistical analysis

Sex-specific characteristics of the study population by smoking status such as age, hemoglobin level, and height were expressed as means ± SD. Sex-specific prevalences of drinking status (daily, often), hypertension, high BMI, low BMI, diabetes, and dyslipidemia were also shown as percentages. Significant differences were evaluated using analysis of variance (ANOVA) for continuous variables and the chi-squared test for proportions.

Logistic regression was used to calculate odds ratios (ORs) and 95% confidence intervals (CIs) for incident height loss and hemoglobin levels and smoking status, respectively. Two different approaches were used to make adjustments for confounding factors. Model 1 adjusted only for age (age-adjusted model). In Model 2 (multivariable model), we included several other potential confounding factors, namely drinking status (none, often, daily), hypertension (yes, no), diabetes (yes, no), dyslipidemia (yes, no), and BMI status (high [≥25.0 kg/m^2^], normal [18–22.9 kg/m^2^, 23.0–24.9 kg/m^2^], low [<18 kg/m^2^]), and in the evaluation of the association between hemoglobin levels and height loss, smoking status (never, former, current) was also included. Although BMI is a variable that includes height, BMI also indicates weight relative to height. Thus, we included BMI status as a confounder in the present analysis.

Values of p<0.05 were regarded as statistically significant. All statistical analyses were performed with SAS for Windows (version 9.4; SAS Inc., Cary, NC, USA).

## Results

### Characteristics of the study population by smoking status

Characteristics of the study population by smoking status are shown in Table 1.

**Table 1:** Characteristics of the study population by smoking status.

	Smoking status			*p*
	Never	Former	Current	
Men				
No. of participants	1,462	2,156	1,900	
Age, years	49.8 ± 8.5	53.0 ± 8.7	50.4 ± 8.2	<0.001
Daily drinker, %	16.3	28.5	30.5	<0.001
Often drinker, %	50.6	52.3	47.4	0.007
Hypertension, %	28.4	35.0	27.0	<0.001
High BMI (BMI ≥ 25 kg/m^2^), %	31.7	35.2	32.3	0.055
Low BMI (BMI < 18 kg/m^2^), %	1.7	0.9	3.0	<0.001
Diabetes, %	7.0	10.4	10.7	<0.001
Dyslipidemia, %	49.0	50.6	52.3	0.168
Hemoglobin, g/dL	15.0 ± 1.0	14.9 ± 1.0	15.3± 1.1	<0.001
Height, cm	170.4 ± 6.2	170.3 ± 5.7	170.6± 5.7	0.186
Women				
No. of participants	2,440	580	446	
Age, years	49.9 ± 8.1	49.2 ± 7.1	48.5 ± 6.6	<0.001
Daily drinker, %	7.0	17.6	22.9	<0.001
Often drinker, %	35.6	46.0	41.0	<0.001
Hypertension, %	14.1	14.8	13.5	0.814
High BMI (BMI ≥ 25 kg/m^2^), %	13.5	14.8	15.9	0.328
Low BMI (BMI < 18 kg/m^2^), %	9.5	9.1	13.5	0.031
Diabetes, %	2.5	3.1	3.1	0.589
Dyslipidemia, %	34.1	30.5	34.1	0.243
Hemoglobin, g/dL	13.0 ± 1.3	13.0 ± 1.2	13.4 ± 1.2	<0.001
Height, cm	157.9 ± 5.6	158.6 ± 5.2	158.4 ± 5.1	0.005

Continuous values are presented as means ± standard deviation. BMI: body mass index. *P* <0.05 was considered significant.

For both men and women, daily drinking was significantly positively associated with smoking status (never, former, current).

Among both men and women, current smokers had significantly higher hemoglobin levels than never smokers. Among men, hemoglobin level (mean ± SD) was 15.3 ±1.1 g/dL among current smokers and 15.0 ±1.0 g/dL among never smokers (p<0.001). Among women, the corresponding values were 13.4 ±1.2 g/dL and 13.0 ±1.3 g/dL (p<0.001).

There were 2,715 former smokers (2,146 men and 569 women) and 2,331 current smokers (1,893 men and 438 women) from whom it was possible to calculate the Brinkman Index. The median (interquartile range) Brinkman Index for former smokers was 360 (200–600) among men and 150 (60–300) among women (p calculated after logarithmic transformation <0.001). Among current smokers, the corresponding values were 540 (400–760) in men and 338 (210–480) among women (p calculated after logarithmic transformation <0.001).

### Characteristics of the study population by height loss status

Table 2 shows the characteristics of the study population by height loss status during the observational period. For both men and women, participants with height loss were significantly older than participants without height loss. Among men, mean age was 50.7 ± 8.5 years for those without height loss and 52.8 ± 8.9 years for those with height loss (p<0.001). Among women, the corresponding values were 48.9 ± 7.3 years and 51.8 ± 8.5 years (p<0.001).

**Table 2:** Characteristics of the study population by height loss status.

	Height loss		*p*
	Absent	Present	
Men			
No. of participants	4,139	1,379	
Age, years	50.7 ± 8.5	52.8 ± 8.9	<0.001
Daily drinker, %	25.2	28.1	0.039
Often drinker, %	50.5	49.2	0.428
Former smoker, %	38.4	41.0	0.095
Current smoker, %	33.9	36.0	0.166
Hypertension, %	28.9	35.2	<0.001
High BMI (BMI ≥ 25 kg/m^2^), %	32.4	36.0	0.012
Low BMI (BMI < 18 kg/m^2^), %	1.7	2.3	0.133
Diabetes, %	8.9	11.9	0.001
Dyslipidemia, %	50.9	50.4	0.733
Hemoglobin, g/dL	15.1 ± 1.0	15.0 ± 1.1	<0.001
Height, cm	170.5 ± 5.8	170.3 ± 5.9	0.320
Women			
No. of participants	2,600	866	
Age, years	48.9 ± 7.3	51.8 ± 8.5	<0.001
Daily drinker, %	10.5	11.8	0.294
Often drinker, %	38.5	36.8	0.394
Former smoker, %	16.8	16.5	0.840
Current smoker, %	13.2	11.8	0.269
Hypertension, %	12.5	18.8	<0.001
High BMI (BMI ≥ 25 kg/m^2^), %	12.9	17.4	<0.001
Low BMI (BMI < 18 kg/m^2^), %	10.5	8.3	0.059
Diabetes, %	2.4	3.6	0.060
Dyslipidemia, %	32.0	38.2	<0.001
Hemoglobin, g/dL	13.0 ± 1.3	13.1 ± 1.3	0.377
Height, cm	158.1 ± 5.4	157.9 ± 5.8	0.420

Continuous values are presented as means ± standard deviation. *P* <0.05 was considered significant.

For both men and women, the prevalence of hypertension and high BMI was significantly higher among participants with height loss. Among men, the prevalence of hypertension was 28.9% for those without height loss and 35.2% for those with height loss (p<0.001). The prevalence of high BMI was 32.4% for those without height loss and 36.0% for those with height loss (p = 0.012). Among women, the corresponding values for hypertension were 12.5% and 18.8% (p<0.001) and for high BMI were 12.9% and 17.4% (p<0.001).

Regarding diabetes, only male participants with height loss had a significantly higher prevalence of diabetes. Among men, the prevalence of diabetes was 8.9% for those without height loss and 11.9% for those with height loss (p = 0.001). Among women, the corresponding values for the prevalence of diabetes were 2.4% and 3.6% (p = 0.060).

### Association between height loss and hemoglobin

Table 3 shows the associations between height loss and hemoglobin. A significant inverse association between height loss and hemoglobin levels was observed among men but not among women. The age-adjusted OR (95% CI) for height loss and a 1-SD increment in hemoglobin (1.0 g/dL for men and 1.3 g/dL for women) was 0.94 (0.89, 0.999) for men and 0.97 (0.90, 1.06) for women. Those associations were unchanged after further adjusting for known confounding risk factors. The fully adjusted OR (95% CI) was 0.92 (0.86, 0.98) for men and 0.96 (0.89, 1.04) for women.

**Table 3:** Associations between height loss and hemoglobin levels.

	1-SD increment in hemoglobin	
	(1.0 g/dL for men and 1.3 g/dL for women)	
	OR (95% CI)	*p*
Men		
No. of participants	5518	
No. of height loss (%)	1379 (25.0)	
Age-adjusted	0.94 (0.89, 0.999)	0.045
Multivariable	0.92 (0.86, 0.98)	0.008
Women		
No. of participants	3466	
No. of height loss (%)	866 (25.0)	
Age-adjusted	0.97 (0.90, 1.06)	0.516
Multivariable	0.96 (0.89, 1.04)	0.359

Multivariable: adjusted for age, drinking status (none, often, daily), smoking status (never, former, current), hypertension, diabetes, dyslipidemia, BMI status (<18 kg/m^2^, 18–22.9 kg/m^2^, 23.0–24.9 kg/m^2^, ≥25.0 kg/m^2^). SD: standard deviation; OR: odds ratio; CI, confidence interval; Height loss: being in the highest quartile of annual height decrease. *P* <0.05 was considered significant.

### Correlation between age and hemoglobin levels

Table 4 shows the sex-specific correlation between age and hemoglobin levels. A slight significantly negative correlation between age and hemoglobin levels was observed among men and a slight significantly positive correlation was observed among women. These correlations remained after further adjusting for known confounding factors.

**Table 4:** Correlation between age and hemoglobin levels by sex.

	Simple correlation	Multivariable		
	r (*p)*	Β	β	*p*
Men				
No. of participants	5,518			
Age	-0.20 (<0.001)	-0.02	-0.20	<0.001
Women				
No. of participants	3,466			
Age	0.16 (<0.001)	0.02	0.11	<0.001

Multivariable: adjusted for age, drinking status (none, often, daily), smoking status (never, former, current), high body mass index (BMI) (≥25.0 kg/m^2^), low BMI (<18 kg/m^2^), hypertension, diabetes, and dyslipidemia. r: simple correlation coefficient. Β: parameter estimate. β: standardized parameter estimate. *P* <0.05 was considered significant.

### Association between height loss and smoking status

The ORs (95% CI) for height loss in relation to smoking status are shown in Table 5. Independent of known risk factors, a significant positive association between height loss and smoking status was observed among men but not among women. With never smokers as the reference group, the fully adjusted OR (95% CI) for height loss was 1.15 (0.98, 1.35) for male former smokers and 1.24 (1.05, 1.46) for male current smokers. For women, the corresponding values were 0.98 (0.79, 1.21) and 0.90 (0.71, 1.16), respectively.

**Table 5:** Associations between height loss and smoking status.

	Smoking status				
	Never	Former		Current	
		OR (95% CI)	*p*	OR (95% CI)	*p*
Men					
No. of participants	1,462	2,156		1,900	
No. of height loss (%)	318 (21.8)	565 (26.2)		496 (26.1)	
Age-adjusted	Ref	1.17 (1.00, 1.37)	0.055	1.26 (1.07, 1.48)	0.006
Multivariable	Ref	1.15 (0.98, 1.35)	0.092	1.24 (1.05, 1.46)	0.011
Women					
No. of participants	2,440	580		446	
No. of height loss (%)	621 (25.5)	143 (24.7)		102 (22.9)	
Age-adjusted	Ref	1.00 (0.81, 1.23)	0.969	0.93 (0.73, 1.19)	0.581
Multivariable	Ref	0.98 (0.79, 1.21)	0.820	0.90 (0.71, 1.16)	0.418

Multivariable: adjusted for age, drinking status (none, often, daily), hypertension, diabetes, dyslipidemia, Body mass index (BMI) status (<18kg/m^2^, 18–22.9kg/m^2^, 23.0–24.9 kg/m^2^, ≥25.0kg/m^2^). OR: odds ratio; CI, confidence interval; Ref: referent; height loss: being in the highest quartile of annual height decrease. *P* <0.05 was considered significant.

### Sensitivity analysis

To assess sensitivity, we performed the main analysis again with height loss defined as being in the highest tertile of annual height decrease. We obtained essentially the same results. In the multivariable model, the OR (95% CI) for height loss and a 1-SD increment in hemoglobin was 0.97 (0.91, 1.03) for men and 1.00 (0.93, 1.08) for women. With never smokers as the reference group, the OR (95% CI) for height loss and former smoker status was 1.07 (0.93, 1.24) and 1.21 (1.04, 1.40) for current smoker status among men. Among women, the corresponding values were 0.92 (0.75, 1.12) and 0.91 (0.72, 1.14), respectively.

## Discussion

The major finding of the present study is that smoking is a significant risk factor for height loss among male workers but not among female workers. In male workers, hemoglobin levels were higher among current smokers (15.3 ± 1.1 g/dL) than among never smokers (15.0 ± 1.0 g/dL) (p<0.001) and hemoglobin levels were inversely associated with height loss.

Our previous study with 6,471 male workers and 3,180 female workers revealed a significant inverse association between hemoglobin levels and height loss, defined as the being in highest quartile of annual height decrease, for male workers but not for female workers; the adjusted OR (95% CI) for height loss and a 1-SD increment in hemoglobin (1.0 g/dL for men and 0.8 g/dL for women) was 0.91 (0.86, 0.97) for men and 1.00 (0.92, 1.08) for women [8].

Those results are compatible with our present finding of a significant inverse association between each 1-SD increment in hemoglobin and height loss only among men. We also found further evidence that smoking status (never, former, and current) is significantly positively associated with height loss among men but not among women. In addition, we also found that current smokers have higher hemoglobin levels than never smokers, regardless of sex.

Chronic smoking is known to induce systemic hypoxia [19]. Hemoglobin plays an important role in oxygen transport [20]. To reduce the influence of hypoxia, elevated hemoglobin levels could be observed among smokers. In the present study, current smokers had higher hemoglobin levels than never smokers.

Although current smokers have higher hemoglobin levels than never smokers, hemoglobin elevation itself might not be a risk factor for height loss because hemoglobin plays an important role in reducing hypoxia caused by smoking. Because smoking could cause lumbar disc degeneration [5] and osteoporosis [6], which is associated with vertebral fracture [7], smoking could induce height loss.

In the present study, hemoglobin levels were significantly inversely associated with height loss among men (Table 3), as in our previous study [8]. We also found a significant positive association between smoking status (never, former, current) and height loss (Table 5). However, those associations were limited to men.

As shown is Table 2, for both men and women, participants with height loss were significantly older than participants without height loss. Aging is a process that changes hematopoietic activity [12]. Therefore, for men, hemoglobin levels were slightly but significantly inversely correlated with age (Table 4).

For women, hemoglobin levels were slightly but positively correlated with age, suggesting that aged women could have a lower risk of having a lower hemoglobin level. Menstruation is a known risk factor of hemoglobin loss among young women. Menopause is a risk factor for osteoporosis [21] and intervertebral disc degeneration [22]. Since osteoporosis and intervertebral disc degeneration are major causes of height loss, menstruation status might confound the association between hemoglobin level and height loss.

The influence of smoking might have been stronger for men than for women since men had a significantly higher Brinkman index than women. This difference might result in sex-specific associations between smoking status and height loss, as shown in Table 5.

**Fig 1:**
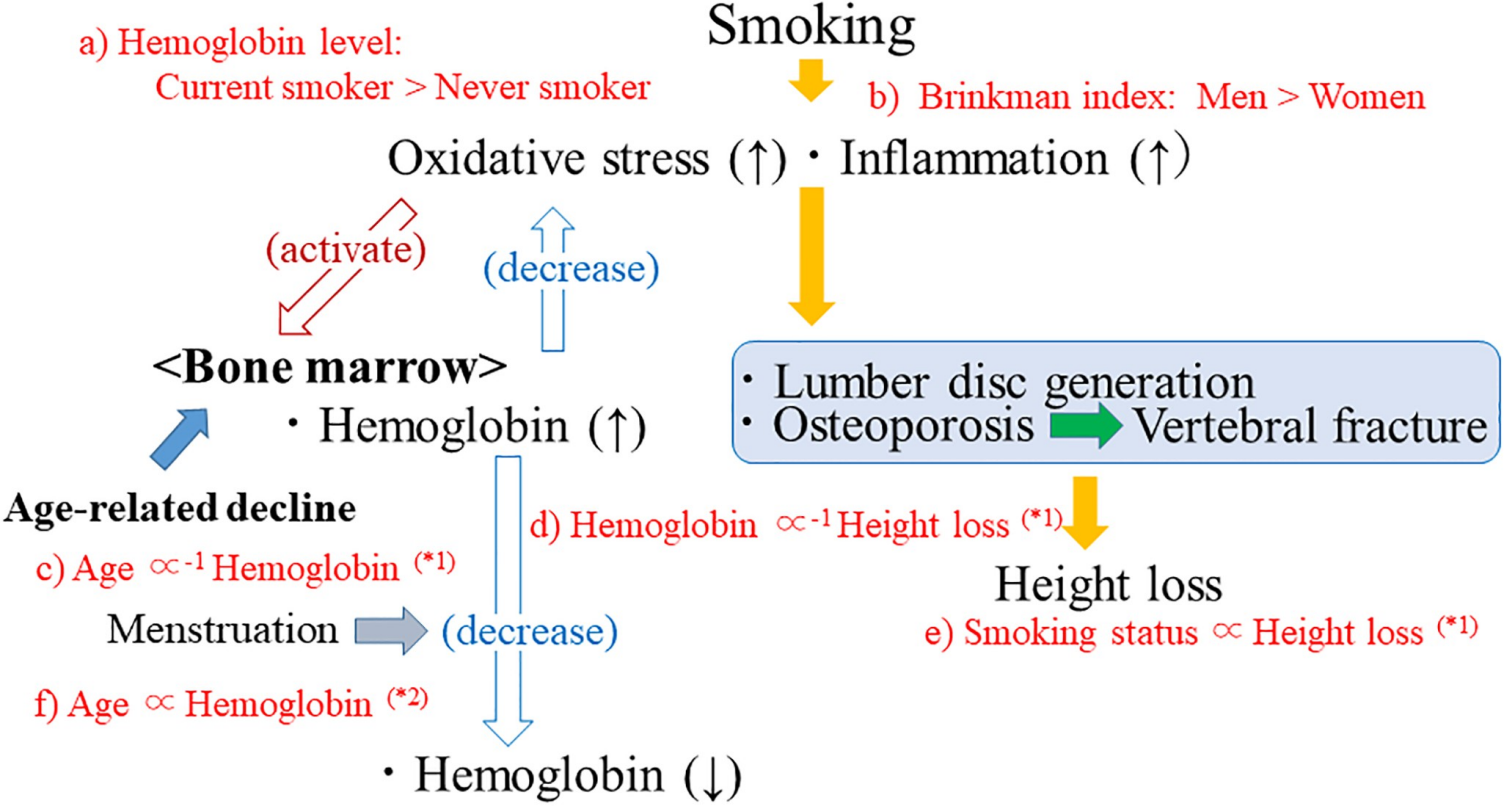
Potential mechanisms underlying the present results. Smoking status is defined as never, former, or current. Associations shown in red (a–f) were observed in the present study. (*1): Observed only among men. (*2): Observed only among women.

Fig 1 shows the potential mechanisms underlying the present results. Associations shown in red (Fig 1a–1f) were observed in the present study. Smoking increases oxidative stress and stimulates inflammation, which causes height loss (Fig 1e). Smoking also activates hematopoiesis and increase hemoglobin levels (Fig 1a). Because men smoke more than women (Fig 1b), the influence of smoking on height loss is stronger in men (Fig 1e). Hematopoiesis activity declines with aging (Fig 1c). However, for women, aging reduces the risk of lower hemoglobin levels because of menopause (Fig 1f). Hemoglobin itself might have a beneficial influence on preventing height loss (Fig 1d). However, menopause might increase hemoglobin levels and increase the risk of osteoporosis [21], which could induce height loss.

Based on our multi-faceted analysis, we were able to determine the potential mechanism underlying the present results because all our present results can be explained by a simple mechanism. We found that smoking is a risk factor for height loss among men. Smoking is associated with higher hemoglobin levels; hemoglobin might prevent height loss. Since height loss starting in middle age is reported to be a risk of increased mortality in later life [1] and smoking is responsible for a considerable proportion of deaths from any cause [4], the present findings help estimate the risk of height loss and death.

The potential limitations of this study warrant consideration. In adults, height loss can be caused by vertebral fractures associated with osteoporosis or intervertebral disc degeneration, for which we did not have available data. Further investigation with data on those diseases is necessary. An efficient cutoff point to define height loss has not been established. In the present study, we used the highest quartile of annual height decrease. However, our sensitivity analysis based on tertiles of annual height decrease showed essentially the same associations. Furthermore, we performed a multi-faceted analysis and the results indicated a simple mechanism. Although hypoxia and oxidative stress might have had a substantial effect on the study results, we had no data to evaluate oxidative stress. Further investigations with markers of hypoxia and oxidative stress such as hypoxia inducing factor (HIF), 8-hydroxydeoxyguanosine (8-OHdG), and superoxide dismutase (SOD) are necessary. As in our previous studies [8–10, 14, 16], no data were available on vertebral fractures and intervertebral disc degeneration, which are known causes of height loss. Most patients with intervertebral disc degeneration and vertebral fractures are asymptomatic [23, 24]. Therefore, plain radiographs, computed tomography, or magnetic resonance imaging is necessary to identify those diseases.

## Conclusions

Smoking is a significant risk factor for height loss in male workers but not in female workers. These findings can help clarify the mechanisms underlying height loss in adults.
